# Theory and Practice of Treatment of Concurrent Major Depressive and Alcohol Use Disorders

**DOI:** 10.1192/j.eurpsy.2022.1153

**Published:** 2022-09-01

**Authors:** A. Samokhvalov

**Affiliations:** Homewood Health Centre, Asu/cpc, Guelph, Canada

**Keywords:** Alcohol use disorder, Concurrent Disorders, major depressive disorder, integrated care

## Abstract

**Introduction:**

Both Major Depressive and Alcohol Use Disorders are highly prevalent. They also are the major contributors to disability and decreased quality of life and, as they are often comorbid with each other, the diagnosis and treatment of concurrent depression and alcohol use disorder represents a challenging task with multiple clinical questions requiring evidence-based recommendations.

**Objectives:**

The goal of this presentation is to review the optimal strategies to treat concurrent alcohol use and major depressive disorders in the context of current research findings and clinical practice.

**Methods:**

Narrative review, knowledge synthesis.

**Results:**

The most up-to-date research findings in the areas of epidemiology of concurrent depression and alcohol use disorder, their differential diagnosis, and treatment approaches will be reviewed. This review will include the current evidence of effectiveness of various antidepressants in treatment of depression concurrent with alcohol use disorder and antidipsotropic agents use for alcohol use disorder in the context of depressive symptoms, as well as their combinations. We will discuss the timeline of initiation of both antidepressants and antidipsotropic agents, non-pharmacological treatment modalities as well as the clinical tools that can be used to properly monitor patients’ progress and optimize the treatment process, and the integrative teamwork necessary to achieve optimal results.

**Conclusions:**

Ultimately, the optimal diagnostic and treatment algorithm and the set of evidence-based treatment recommendations will be presented.

**Disclosure:**

No significant relationships.

